# Mannoside-Modified Branched Gold Nanoparticles for Photothermal Therapy to MDA-MB-231 Cells

**DOI:** 10.3390/molecules25081853

**Published:** 2020-04-17

**Authors:** Han-Chen Lin, Keng-Fang Hsu, Chiao-Ling Lai, Tzu-Chien Wu, Hui-Fen Chen, Chian-Hui Lai

**Affiliations:** 1 Department of Anatomy, School of Medicine, College of Medicine, Kaohsiung Medical University, Kaohsiung 807, Taiwan; hanchen@kmu.edu.tw; 2 Department of Medical Research, Kaohsiung Medical University Hospital, Kaohsiung 807, Taiwan; 3 Department of Medicinal and Applied Chemistry, Kaohsiung Medical University, Kaohsiung 807, Taiwan; hsukf1223@gmail.com; 4 Graduate Institute of Biomedical Engineering, National Chung Hsing University, Taichung 402, Taiwan; devil1012asd1313@gmail.com (C.-L.L.); yingyingwu0610@gmail.com (T.-C.W.)

**Keywords:** branched gold nanoparticles, photothermal therapy, near-infrared (NIR) laser, MDA-MB-231 cell, mannose receptor

## Abstract

Recently, gold nanoparticles (Au NPs) have been used to study the treatment of malignant tumors due to their higher biocompatibility and lesser toxicity. In addition, they can be excited through a specific wavelength to produce oscillating plasmonic photothermal therapy (PPTT) on the basis of the localized surface plasma resonance (LSPR) effect. Au NPs can be heated to kill cancer cells in specific parts of the body in a noninvasive manner. In this study, branched gold nanoparticles (BAu NPs) were prepared by mixing HAuCl_4_ in a 4-(2-hydroxyethyl)-1-piperazineethanesulfonic acid (HEPES) buffer solution in a molar ratio of 1:2000. The UV–vis absorption peak was detected in the range of 700–1000 nm. Subsequently, BAu NPs were chemically linked to a thiol-modified mannoside molecule via a stable sulfur–Au covalent bond (Man@BAu NPs). Due to the presence of abundant mannose receptors on human-breast-cancer cells, MDA-MB-231, Man@BAu NPs were found to be abundant inside cancer cells. After irradiating the Man@BAu NP-laden MDA-MB231 switch with a near-infrared (NIR) laser at 808 nm wavelength, the photothermal-conversion effect raised the surface temperature of Man@BAu NPs, thus inducing cell death. Our experiment results demonstrated the advantages of applying Man@BAu NPs in inducing cell death in MDA-MB-231.

## 1. Introduction

Malignant tumors are one of the main causes of human deaths in the past decade [1]. The medical treatment of malignant tumors is usually through surgical resection, radiotherapy, or chemotherapy. However, most chemotherapeutic drugs are not specific to tumor cells. Indeed, these nonspecific chemotherapeutic drugs can inhibit tumor growth, but also cause harm to other cells. This unspecific targeting is associated with side effects such as immunity weakening, hair loss, and vomiting. An effective targeted-therapy method, combined with laser-induced hyperthermia therapy [2,3,4], could be an alternative way to treat solid tumors. Under specific light irradiation, plasmonic photothermal therapy (PPTT) can apply a photoabsorber to localize heat and therefore focus on the target site to kill cancer cells at a specific part of the body in a noninvasive manner.

**Figure 1:**
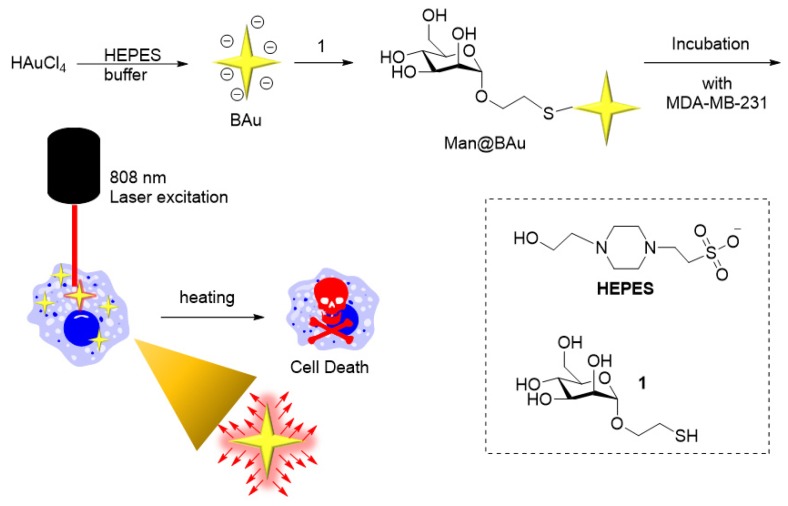
Experiment concept. BAu was prepared by mixing HAuCl_4_ in a HEPES buffer solution. BAu NPs were chemically linked to a thiol-modified mannoside **1** via a stable sulfur–Au covalent bond. After irradiating the Man@BAu NP-laden MDA-MB231 switch with a near-infrared (NIR) laser at 808 nm wavelength, the photothermal-conversion effect raised the surface temperature of Man@BAu NPs, thus inducing cell death.

**Figure 2:**
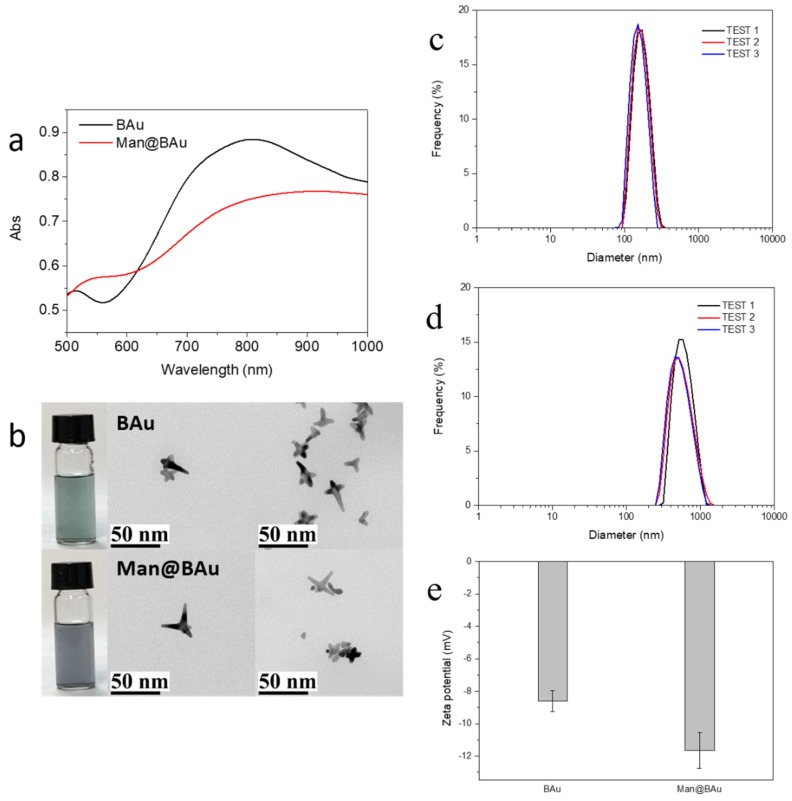
(**a**) UV–vis absorption spectra and (**b**) TEM images of branched gold (BAu) and mannose-modified Bau (Man@BAu). Dynamic-light-scattering (DLS) size distributions of (**c**) BAu and (**d**) Man@BAu NPs; (**e**) zeta-potential of BAu and Man@BAu NPs.

Recently, various gold nanoparticles (Au NPs) have been used to study the treatment of malignant tumors due to their high biocompatibility and low toxicity [1,5,6]. Moreover, Au NPs have excellent localized surface-plasma-resonance (LSPR) property, which makes them good material for PPTT [4,7,8]. In particular, near-infrared (NIR, λ = 650–1350 nm) laser-induced PPTT has received much attention since NIR light can penetrate biological tissue with lower energy absorption; hence, it is the NIR biological window [9,10]. PPTT theory is based on energy conversion; NIR light is converted into heat energy, resulting in high temperatures, which has a good inhibitory effect on cancer cells. The rationale of hyperthermia is direct cell killing or inducing cell apoptosis at ~43 °C [11]. According to a previous report, Au NPs with sharp tips have higher efficiency for photothermal conversion than other shapes do [12]. Therefore, branched gold nanoparticles (BAu NPs) are prepared according to a previously published method [13]. Gold(III) tetrachloride salt is added to a 2-[4-(2-hydroxyethyl)-1-piperazinyl]ethanesulfonic acid (HEPES) buffer to form BAu NPs within an absorption peak with a broad frequency between 700 and 1100 nm in the ultraviolet–visible spectrum (Figure 1 and Figure 2) in which the wavelength is an NIR area. As long as the laser of this NIR wavelength range is used, BAu NP irradiation has the heating effect. 

Attaching an active targeting molecule on the NP is an important strategy for enhanced uptake in specific cells [1,14]. Recent studies on cancer cells identified the presence of mannose receptors on the surface of MDA-MB-231, which is a human-breast-cancer cell line [15,16,17]. In order to increase the selectivity to breast-cancer cell line MDA-MB-231, we first modified BAu NPs with thiol-modified mannoside 1 [18] to yield Man@BAu NPs through forming a stable S–Au covalent bond (Figure 1). Since mannose receptors are abundantly present on human-breast-cancer cell line MDA-MB-231, it was expected that Man@BAu NPs would easily be taken up by the cancer cells [15,16,17]. It was anticipated that Man@BAu NPs would penetrate MDA-MB-231 via mannose-dependent endocytosis. It is an active targeting strategy by attaching a targeting molecule onto a nanoparticle. Some researchers also used carbohydrate modification on nanoscaffolds for targeting purposes [19,20,21,22,23]. After irradiation by a near-infrared (NIR) laser at 808 nm wavelength, the photothermal-conversion effect raises the surface temperature of the nanoparticles and destroys the cancer cells. 

## 2. Results

### 2.1. BAu and Man@BAu Synthesis

BAu NPs were synthesized by reducing gold(III) tetrachloride salt with HEPES at room temperature for 60 min (Figure 1) [13]. In order to achieve a good heating-conversion effect, it was necessary to develop the best condition for synthesizing BAu NPs that could meet the maximal absorption value at the NIR wavelength. Factors such as the concentration of the HEPES buffer solution, pH value, and the reaction molar ratio of HEPES to gold(III) tetrachloride salt were all considered [13,24]. The pH value of the HEPES buffer solution was to affect the size of the BAu NPs (Figure S1) [24,25]. The molar ratios of HEPES to the Au(III) salt also played an important role in the formation of BAu NPs [13]; the higher ratios showed a lower wavelength because the HEPES high concentration could rapidly assemble into structures, blocking the growth of branches (Figure S2). An earlier study [13] showed that mixing HEPES with HAuCl_4_ at a ratio of 3000:1 while adjusting the concentrations of HEPES could result in a morphological change of BAu NPs. Therefore, in the present study, the ratio of HEPES to HAuCl_4_ was kept at 2000:1 while examining the effect of different buffer concentrations of HEPES (2 M, 1 M, and 0.1 mM). The self-assembly behavior was more pronounced at higher concentrations of the HEPES buffer (Figure S3). The best condition for the of synthesis of BAu NPs was determined to be 2 M HEPES buffer (pH 7.2), and mixed with HAuCl_4_ at the ratio of 2000:1. Man@BAu NPs were then synthesized through a typical ligand-exchange method [26,27]. The chemisorbed bilayer HEPES molecules on the BAu NPs surface were replaced with thiolated mannoside **1** (Please see Scheme S1 for the detailed synthetic procedure of **1,** and Figure S4 for the NMR spectrum of **1** in the SI) through a ligand-exchange reaction, and then the corresponding Au–S bond formation to give Man@BAu (Figure 1). Despite this small size, BAu NPs supported LSPR centered at ~810 nm (Figure 2a) within the biologically transparent NIR spectral window because of its multibranched shape. By controlling the ratio of Au(III) salt to HEPES, we produced BAu NPs with average sizes of ~18.7 nm (Figure 2b and Figure S5). The morphology of the Man@BAu NPs were also observed by TEM, and results are shown in Figure 2b. Man@BAu NPs were uniform, with average sizes of ~16 nm. The slightly reduced size of the branch of Man@BAu could have been contributed by reducing agent TCEP reacting with BAu. TCEP was applied during the synthetic procedure for Man@BAu NPs. The peak broadness of Man@BAu was likely due to heterogeneity in the lengths and diameters of the branches. The UV−vis spectrum of the BAu solution showed a strong broad peak in the range of 700−1000 nm, which was a result of longitudinal plasmon resonance (LPR), which is due to the high aspect ratio of the branches. This broad NIR absorption of BAu NPs makes them excellent candidates for photothermal therapy, and broad absorption (rather than sharp narrow absorption) also reduces the need for a specific laser frequency. The LPR of Man@BAu NPs, determined by the UV−vis absorption spectrum (Figure 2a and Figure S5) was broader and centered at 800–900 nm, matching well with the 808 nm laser used in this work for an efficient photothermal effect. Hydrodynamic-diameter distributions were also determined by DLS; BAu and Man@BAu NPs were 154.3 ± 36.8 and 597.5 ± 172.6 nm with a corresponding polydispersed index (PDI) of 0.31 and 0.48, respectively (Figure 2c,d). The zeta potential of BAu and Man@BAu NPs was determined to be −8.6 ± 0.7 and −11.7 ± 1.1 mV, respectively (Figure 2e). BAu and Man@BAu NPs were also subjected to FTIR analysis (Figure S6). The FTIR spectra of the HEPES-stabilized BAu NPs and free HEPES molecules were collected as powder samples. The FTIR spectrum of the HEPES showed several distinct peaks at 1044, 1183, 1222, and 1460 cm^−1^ corresponding to the piperazine ring stretching or −O−H rocking, −C−N bond stretching, −S=O bond stretching, and −CH_2_ scissoring, respectively [13]. The band at 1635 cm^−1^ of the BAu NPs could be attributed to the reaction product of oxidized products of HEPES forming during the reduction of Au^3+^ [28]. We identified functional mannoside groups in the FTIR spectrum that included the vibration bands of methoxyl groups at 1411 cm^−1^. In addition, the strong stretch at 1130–1066 cm^−1^ corresponded to C–O–C linkage, and the middle unsymmetrical shrinkage at 3000–2850 cm^−1^ represented the -CH_2_-cyclopentane group [29]. For the FTIR spectrum of Man@BAu NPs, since BAu NPs were stabilized by HEPES, those peaks at 1044, 1198, and 1454 cm^−1^ were from vibration bands of HEPES. In addition, several distinct peaks could be seen at 1133, 1379, and 2844 cm^−1^ that mannoside contributed. Thus, we used FTIR spectroscopy to demonstrate that BAu NPs were successfully functionalized with mannoside **1** groups.

### 2.2. Photothermal Performance of BAu and Man@BAu

**Figure 3:**
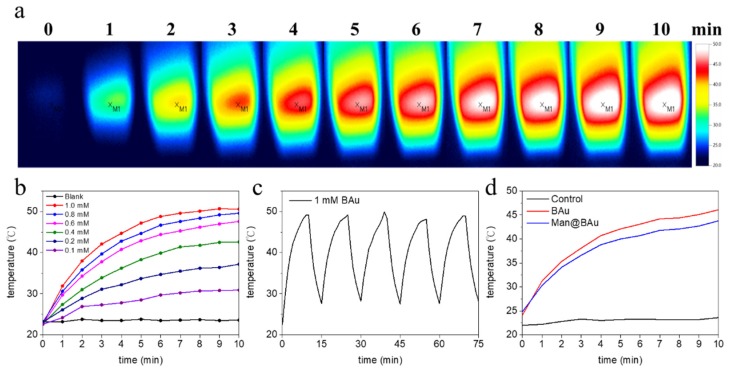
(**a**) Thermal-change images of 1 mM BAu NPs (2 mL) in cuvette using 808 nm laser for 10 min. (**b**) Photothermal effects of BAu NPs at various concentrations under same conditions as in (**a**). (**c**) Temperature variation of BAu NPs during five irradiation cycles (irradiation with 808 nm laser at 1.375 W·cm^−2^ for 10 min and cooled naturally for 5 min). (**d**) Photothermal effects of Man@BAu, BAu, and control (water) under irradiation of 808 nm laser at 1.375 W·cm^−2^ for 10 min.

To investigate the photothermal performance of BAu and Man@BAu NPs, the temperature of the BAu NP solution was recorded under the irradiation of an 808 nm laser at 1.375 W·cm^−2^ for 10 min (Figure S7 for laser instrument). As a comparison, the photothermal effect of the BAu NPs, Man@BAu NPs, and ultrapure water was performed under identical conditions. All results are shown in Figure 3. The temperature changes of BAu NPs and Man@BAu NPs were 28 and 26 °C, respectively. Compared to the temperature of the control (ultrapure water), it remained at the same value within 10 min under the irradiation of an 808 nm laser. BAu and Man@BAu NPs exhibited higher photothermal performance than that of ultrapure water (Figure 3d). Moreover, BAu NPs could still exhibit observable photothermal performance, even at low concentration (0.1 mM, Figure 3b), and an increment of 28 °C could be achieved in 10 min under irradiation. The photothermal-conversion efficiency of BAu NPs was measured with a previously published method [3]. The temperature change of BAu NPs (1 mM) was recorded as a function of time under the continuous irradiation of 808 nm laser at 1.375 W·cm^−2^ for 10 min, and cooled naturally for 5 min during five irradiation cycles (Figure 3c). BAu NPs (1 mM) exhibited the highest temperature elevation of 28 °C under irradiation for 10 min. Photothermal-conversion efficiency η was calculated using Equation (1).

(1) $$\eta =\frac{hS\left(T_{max}-T_{sur}\right)}{I\left(1-10^{-A_{808}}\right)}$$

where h is the heat-transfer coefficient, S is the surface area of the container, and the value of hS was obtained from Equation (S3) (in Supplementary Materials). T_max_ is the equilibrium temperature, T_surr_ is the ambient temperature of the surroundings, and (T_max_ − T_surr_) was 28 °C according to Figure 3c. Q_dis_ expresses heat dissipated from light absorbed by the quartz sample cell itself, and it was measured independently to be 7.9 mW using a quartz cuvette cell containing pure water without NPs. I is the incident laser power (1.375 W·cm^−2^), A_808_ is the absorbance (1.836) of BAu NPs at 808 nm (Figure S8a). Thus, the 808 nm laser heat-conversion efficiency (η) of the BAu NPs could be calculated to be 27.3%.

The photothermal stability of BAu NPs was also evaluated. As shown in Figure 3c, BAu NPs showed excellent stability after five cycles of irradiation, which illustrated that BAu NPs are very suitable for photothermal applications, even compared with other studies that experimentally investigated the photothermal-conversion efficiencies of Au NPs of varying shapes and sizes [6,30,31,32,33,34,35]. The η values from some of these reports were from 22% to 100%. The absorption cross-section and LPR peak position were shape-dependent, and therefore might affect light-to-heat conversion and photothermal-conversion efficiencies.

### 2.3. BAu and Man@BAu Stability

**Figure 4:**
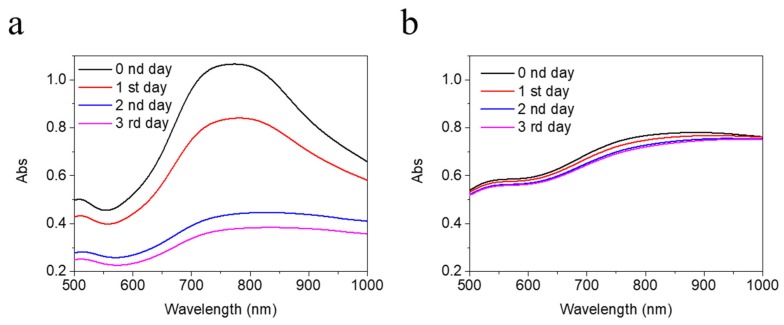
UV absorption of 0.5 mM (**a**) BAu and (**b**) Man@BAu NPs in 150 mM HEPES for 1 to 3 days for testing their stability. UV spectrum of Man@BAu NPs showed more stability.

In order to make use of Man@BAu NPs in cell experiments, it was necessary to suspend NPs in a cell-culture medium. Thus, the stability of Man@BAu in a 150 mM isotonic solution was analyzed (Figure 4). It was demonstrated that most ions in a solution exhibit weak-to-almost-no affinity to AuNP. The impact of the physical stability to AuNP can be achieved through adjusting the ionic strength of the solution, and the double-layer composition and thickness [36]. In the case of BAu (Figure 4), because the anion concentration of HEPES was lowered from 2 M to 150 mM, the adsorbed anions on the BAu surface were easily desorbed as a result of instability and aggregation. As shown in Figure 4a, the measured UV intensity of BAu decreased as time increased. By contrast, Man@BAu showed almost a constant value in the UV spectrum (Figure 4b). It was likely that the mannoside moiety on the surface of Man@BAu could provide stability in the isotonic solution because of the stable S–Au covalent bond on Man@BAu, negative charge, or steric stability. Electrostatic and steric stabilization could be combined to maintain nanoparticle stability in the solution. This kind of stabilization is generally provided by means of ionic surfactants [37]. The phenomenon was also found in a report [36] that a 6-mercaptohexanoic acid-functionalized Au nanostar formed self-assembled monolayers (SAMs) on the Au surface; thus, the SAM-stabilized structures were electromagnetically coupled. The higher stability of Man@BAu could be contributed by both electrosteric stabilizations.

### 2.4. Fluorescence Microscopy Study and Cytotoxicity Assay

**Figure 5:**
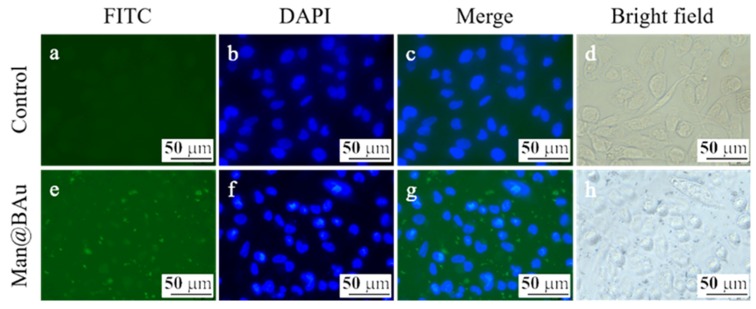
Fluorescent microscopic images of MDA-MB-231 cells (**a**–**d**). MDA-MB-231 cells incubated with FITC-Man@BAu (green, **e**). Nucleus was labeled with DAPI (blue) (**f**), merge (**g**), bright field (**h**).

**Figure 6:**
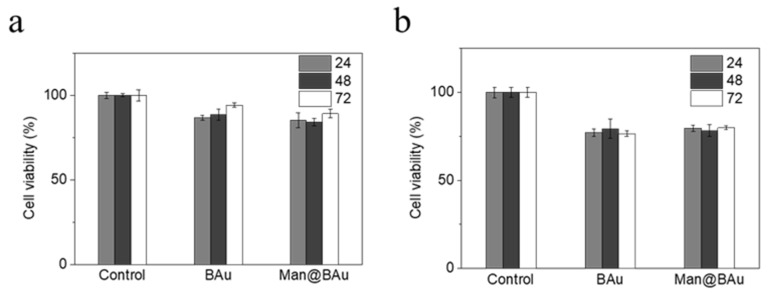
BAu or Man@BAu of (**a**) 0.05 mM and (**b**) 0.1 mM cultured with MDA-MB-231 cell for 24, 48, 72 hours, respectively, to test for cytotoxicity by MTT assay.

The recognition of Man@BAu NPs by MDA-MB-231 cells was investigated by fluorescence microscopy. In order to see the Man@BAu in fluorescence microscopy, the FITC-labeled mannoside-modified branched gold nanoparticles (FITC-Man@BAu NPs) were first prepared (please see Figure S9 in SI for the UV absorption spectrum of FITC-Man@BAu NPs). Briefly, during the synthetic procedure of FITC-Man@BAu, FITC-PEG-SH and thiol-modified mannoside **1** were added together with TCEP and then mixed with BAu NPs to obtain FITC-Man@BAu. MDA-MB-231 cells were seeded at a density of 3 × 10^4^ cells/well in a 96-well culture plate, and then incubated with FITC-Man@BAu NPs at a concentration of 0.05 mM for 0.5 h. Cells in the culture plate were washed with PBS containing 0.1% PEG-200 three times to remove the nonuptake of FITC-Man@BAu NPs; then, cells were fixed 4% PFA. As shown in Figure 5, MDA-MB-231 cell nuclei were stained by DAPI as shown in blue, and FITC-Man@BAu NPs are shown as fluorescent green in the images, in which FITC-Man@BAu NPs had effective endocytosis by MDA-MB-231 cells (Figure 5g). Before conducting a PTT experiment in the cell, the cytotoxicity of BAu and Man@BAu to MDA-MB-231 cells was examined by MTT assays at 37 °C for 24, 48, and 72 hours. As shown in Figure 6a, in a concentration of 0.05 mM, cells remained ~90% viable, indicating the low cytotoxicity of BAu and Man@BAu NPs. The viability of the cells was 80% when exposed to 0.1 mM of either NP (Figure 6b).

### 2.5. Laser-Irradiation Experiment and Cell-Killing Effect

**Figure 7:**
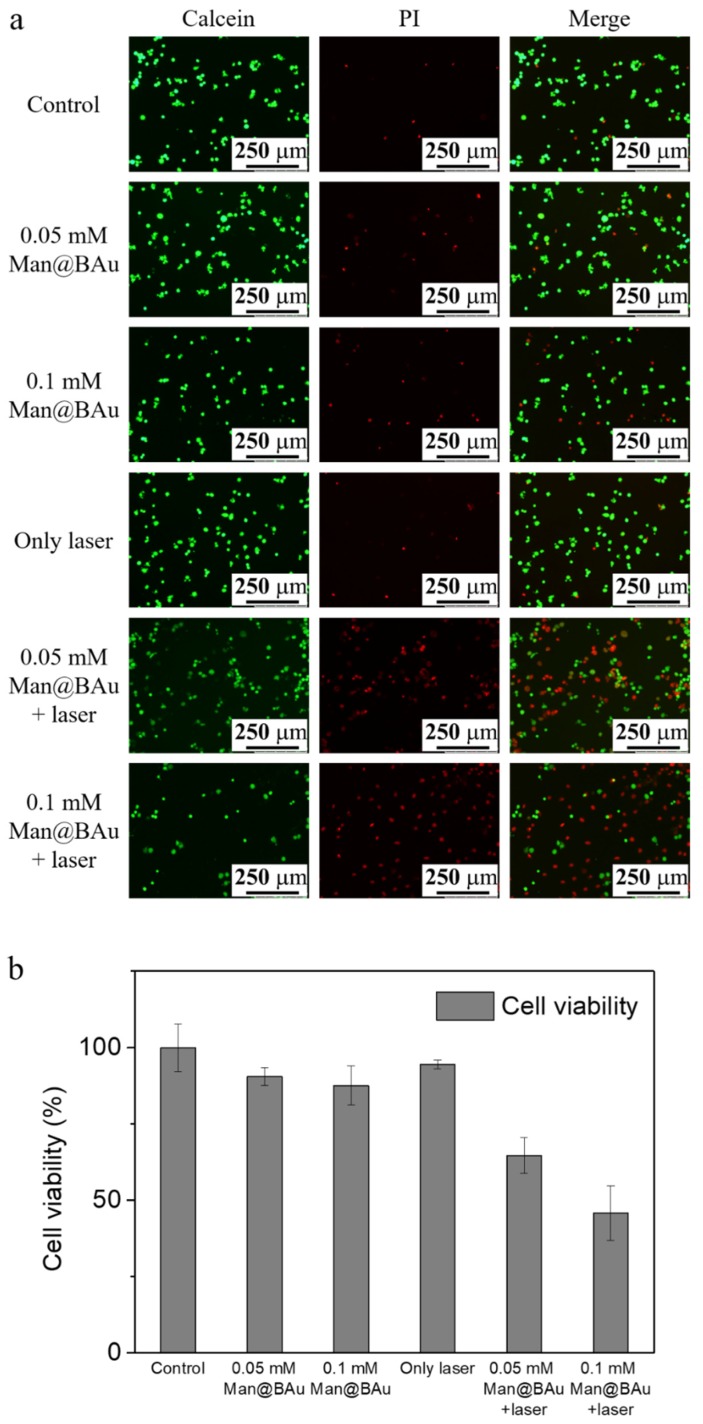
MDA-MB-231 cell incubated with 0.05 or 0.1 mM Man@BAu NPs for 30 min, followed by being irradiated by 808 nm laser for 10 min to observe cell viability. (**a**) Live/dead kit images presented green for viable cells and red for apoptotic cells. (**b**) MTT data presented that cell-killing effect for 0.05 mM was about 36%, and about 55% for 0.1 mM. Cell viabilities of MDA-MB-231 cells exposed to various concentrations of Man@BAu NPs with and without the 808 nm laser analyzed using MTT assay (n = 6).

Photothermal ablation of MDA-MB-231 cells after exposure to various concentrations of Man@BAu NPs was performed using the 808 nm laser. MDA-MB-231 cells were seeded at a density of 2 × 10^4^ cells/well in a 96-well culture plate and then incubated with Man@BAu NPs for 0.5 h. Subsequently, cells were irradiated with the 808 nm laser for 10 min. Experiments were divided into six groups: cells alone, cells treated with Man@BAu NPs at 0.05 and 0.1 mM, cells treated with the 808 nm laser, cells treated with Man@BAu NPs at 0.05 and 0.1 mM, and using the 808 nm laser. Cells exposed to Man@BAu NPs without laser irradiation showed high viability. Cells that were irradiated by using the 808 nm laser without incubated Man@BAu NPs also showed high viability. Cell deaths were noticed when MDA-MB-231 cells were irradiated after exposure to either 0.05 or 0.1 mM Man@BAu (Figure 7a). The viabilities of MDA-MB-231 cells exposed to Man@BAu NPs with different treatments were also assayed by MTT. When the concentration of Man@BAu NPs was at 0.05 mM, after 808 nm laser irradiation, the percentage of dead cells was ~36%. When the concentration of Man@BAu NPs increased to 0.1 Mm, the percentage of dead cells was ~55% (Figure 7b).

## 3. Materials and Methods 

### 3.1. Materials and Apparatus

Gold(III) chloride hydrate (HAuCl_4_3H_2_O, Alfa Aesar), 2-[4-(2-hydroxyethyl) piperazin-1-yl] ethanesulfonic acid (HEPES, Acros), tris(2-carboxyethyl)phosphine hydrochloride (TCEP, Acros), and fluorescein PEG thiol (FITC-PEG-SH PG2-FCTH-1k, NANOCS INC, Boston, USA) were received without further purification. Then, a 2 M HEPES buffer solution was prepared by dissolving the HEPES powder in double-deionized water (dd-H_2_O), then adjusted to pH 7.2 by adding NaOH at 20 °C. The 150 mM isotonic HEPES solution was prepared by diluting the 2 M buffer solution with dd-H_2_O, and the pH of the solution was adjusted to 7.4. The absorption spectra of BAu NP and Man@BAu were measured using a UV–vis NIR spectrophotometer (JASCO V-730, Tokyo, Japan). A JEOL 2100 Transmission electron microscope (TEM) was used for imaging the particles (JEOL JEM-2100, Tokyo, Japan). For TEM sample preparation, a drop of a 10 μL sample was left to rest on Formvar/carbon 200 mesh copper grids for three days in a vacuum. The hydrodynamic diameter of the BAu or Man@BAu was analyzed by dynamic light scattering (DLS, HORIBA SZ-100, Kyoto, Japan) at 25 °C. The surface chemistry of the nanoparticles was studied by Fourier transform infrared spectroscopy (FTIR; BRUKER ALPHA, Ettlingen, Germany). Cell viability and cytotoxicity were measured by using an ELISA reader (Bio Tek Synergy H1, Winoovaki, VT, USA). Fluorescence images were taken with an inverted fluorescence microscope (Leica DMi8, Wetzlar, Germany).

### 3.2. Preparation of Branched Gold Nanoparticles (BAu NPs)

We synthesized 0.5 mM BAu NPs according to a previously reported method [13]. Then, 10 μL of HAuCl4 (0.5 M in dd-H_2_O) was added to a stirring solution of 5 mL of 2 M HEPES (pH 7.2). The resulting mixture was stirred for 60 min, allowing Au(III) to reduce completely (color turned dark blue). The supernatant was discarded after centrifugation at 12,000 rpm for 10 min three times. The precipitate was redissolved to 3 mL in 150 mM HEPES (pH 7.4).

### 3.3. Preparation of Mannose-Modified Branched Gold Nanoparticles (Man@BAu NPs)

We added 0.4 mL of thiol-modified mannoside (2.5 mg/mL in dd-H_2_O) to a 0.6 mL of freshly prepared TCEP aqueous solution (0.83 mg/mL) in a 4.0 mL vial for 30 min. A solution of 1.5 mL BAu NPs (1.0 mM) was then added to the vial under a nitrogen atmosphere. The reaction was stirred for 30 min, and 0.5 mL of 2 M HEPES (pH 7.2) was added to become 0.5 mM of Man@BAu NPs. The supernatant was discarded after centrifugation at 12,000 rpm for 10 min three times, and the precipitate was redissolved in 150 mM HEPES (pH 7.4).

### 3.4. Preparation of FITC-Labeled Mannoside-Modified Branched Gold Nanoparticles (FITC-Man@BAu NPs)

We added 0.4 mL of thiol-modified mannoside 1 (2.5 mg/mL in dd-H_2_O) and 16.7 μL of FITC-PEG-SH (25 mg/mL in dd-H_2_O) to 0.6 mL of freshly prepared TCEP aqueous solution (0.83 mg/mL) in a 4.0 mL vial for 30 min. Then, 1.5 mL of BAu NPs (1.0 mM) was added to the vial under a nitrogen atmosphere. The reaction was stirred for 30 min, and 0.5 mL of 2 M HEPES (pH 7.2) was added to form 0.5 mM of FITC-Man@BAu NPs. The supernatant was discarded after centrifugation at 12,000 rpm for 10 min three times, and the precipitate was redissolved in 150 mM HEPES (pH 7.4).

### 3.5. Temperature-Elevation Profile Upon 808 nm Laser Irradiation

BAu NPs (1 mL) with different Au ion concentrations in the 150 mM HEPES buffer were placed into a 3 mL quartz cuvette. The photoinduced temperature changes of the solutions were achieved under irradiation by infrared diode laser at 808 nm with a fixed beam size of 80 mm^2^ for 10 min. Temperature changes were recorded by thermal imaging camera (Testo 868, Lenzkirch, Germanry) after 808 nm laser irradiation.

### 3.6. Cell-Culture Conditions

MDA-MB-231 (human-breast-cancer-cell-line) cells from BCRC (Bioresource Collection and Research Center, Hsinchu, Taiwan) were cultured in Dulbecco’s Modified Eagle Medium (DMEM) supplemented with 10% fetal bovine serum (FBS) and 1% penicillin–streptomycin–neomycin solution. Cells were maintained at 37 °C in a humidified atmosphere containing 5% CO_2_.

### 3.7. Cellular-Uptake Image Studies

For the cellular uptake of the FITC-Man@BAu experiments, MDA-MB-231 cells were seeded at a density of 3 × 104 cells/well in a 96-well culture plate for 24 h. Subsequently, the culture medium was replaced with a fresh medium containing 0.05 mM of FITC-Man@BAu NP. After 30 mins of incubation, cells were washed with 0.1% PEG-200 dissolved in a PBS solution three times, and then fixed with 4% paraformaldehyde (PFA) solution for 10 min at room temperature. Cells were further washed by PBS for three times. Afterwards, the nucleus was stained with a DAPI (4′,6-diamidino-2-phenylindole) (0.1 µg/mL) for 10 min. All samples were observed by using an inverted fluorescence microscope (Leica DMi8, Wetzlar, Germany)

### 3.8. Photothermal Effect of Man@BAu NPs in MDA-MB-231 Cells

MDA-MB-231 cells were seeded at a density of 2 × 104 cells/well in a 96-well culture plate and incubated at 37 °C for 24 h, followed by treatment with Man@BAu or BAu for 0.5 h. Subsequently, the medium containing the noninternalized BAu was discarded, and cells were washed with 0.1% PEG-200 of PBS three times and respectively irradiated with an 808 nm diode laser (1.08 W) for 10 min. Live cells were stained with calcein in green and dead cells, with propidium iodide (PI) in red. The fluorescence images of live and dead cells were observed by using an inverted fluorescence microscope. To quantitatively evaluate the effect of the 808 nm laser on MDA-MB-231 cells, MTT (3-(4,5-Dimethylthiazol-2-yl)-2,5-diphenyltetrazolium bromide) assay was also carried on Man@BAu at 0.05 mM. After cells were incubated for 0.5 h, each well was replaced, and cells were washed three times with 0.1% PEG-200 of PBS (pH 7.4). Photothermal treatment was then performed using an 808 nm laser. Cells were exposed under the 808 nm laser for 10 min before adding 100 µL of the MTT solution (0.4 mg/mL). After incubating for 1 h more, the medium containing MTT was carefully removed from each well, and DMSO (100 µL) was added to each well to dissolve the purple crystals. The plates were gently shaken for 10 min at room temperature before measuring absorbance at 570 nm with an ELISA reader (BioTek, Winoovaki, VT, USA).

## 4. Conclusions

In summary, we successfully prepared mannoside-modified branched-gold nanoparticles (Man@BAu NPs) with high stability in an isotonic solution. Man@BAu NPs had low cytotoxicity. The UV–vis absorbance spectrum of Man@BAu NPs had broad NIR absorption in the biological window, which showed good photothermal conversion when it was irradiated by the 808 nm laser. These Man@BAu NPs had endocytosis by MDA-MB-231 cells within 0.5 h. Lastly, in vitro studies demonstrated that Man@BAu NPs could effectively kill MDA-MB-231 under 808 nm laser irradiation. Although only few materials and conditions were examined in the present study, Man@BAu NPs are a promising material for future applications in photothermal cancer therapy.

## Supplementary Materials

The following are available online. Figure S1: The absorption spectra of the three kinds of different HEPES buffer pH values, Figure S2. The absorption spectra of the different ratios of HEPES to HAuCl_4_, Figure S3: The absorption spectra of the different concentration of HEPES, Figure S4: The NMR of thio-mannoside **1**, Figure S5: (a,b) Shows the fresh prepared solution of BAu and TEM images and (c,d) was 2 days after. The entry size change from ~18.7 nm to ~17.5. (e,f) shows the the fresh prepared solution of Man@BAu and TEM images and (g,h) was 2 days after. The average size maintains at ~16 nm; Figure S6: FTIR spectrum of (a) BAu and (b) Man@BAu NPs, Figure S7: The 808 nm laser instrument, Figure S8: (a) UV absorption spectrum of Man@BAu at the concentration of 1.0 mM. The dash line is absorbance value at 808 nm. (b) A linear plot of time (after 600 s) versus negative natural logarithm of dimensionless driving force temperature obtained from the cooling stage of curve, Figure S9: The UV spretum of FITC-Man@BAu NPs. Scheme S1: The synthetic procedure of mannoside **1**.
