# Supplementary material for: Mannoside-Modified Branched Gold Nanoparticles for Photothermal Therapy to MDA-MB-231 Cells
Source: Molecules. 2020 Apr 17;25(8):1853. doi: 10.3390/molecules25081853 (PMC7221875; doi:10.3390/molecules25081853)
Supplement: Supplementary file 1 [file molecules-25-01853-s001.pdf]

molecules

Supporting Information

Mannoside-Modified Branched Gold Nanoparticles for Photothermal Therapy to MDA-MB-231 Cells

Han-Chen Lin [1,2,†], Keng-Fang Hsu [3,†], Chiao-Ling Lai [4], Tzu-Chien Wu [4], Hui-Fen Chen [3,*] and Chian-Hui Lai [3,4,*]

[1] Department of Anatomy, School of Medicine, College of Medicine, Kaohsiung Medical University, Kaohsiung 807, Taiwan; hanchen@kmu.edu.tw (H.-C.L.)
[2] Department of Medical Research, Kaohsiung Medical University Hospital, Kaohsiung 807, Taiwan
[3] Department of Medicinal and Applied Chemistry, Kaohsiung Medical University, Kaohsiung 807, Taiwan; hsukf1223@gmail.com
[4] Graduate Institute of Biomedical Engineering, National Chung Hsing University, Taichung 402, Taiwan; devil1012asd1313@gmail.com (C.-L.L.); yingyingwu0610@gmail.com (T.-C.W.)
* Correspondence: hfchen@kmu.edu.tw (H.-F.C.); chianhuilai@dragon.nchu.edu.tw(C.-H.L.)
† These authors contributed equally to this work

Table S1.

Entry	2 M HEPES (mL)	HEPES pH-value	0.5 M $HAuCl_4$ (μL)	Peak (nm)
1	4	6.8	8	540
2	4	7.0	8	781
3	4	7.2	8	805

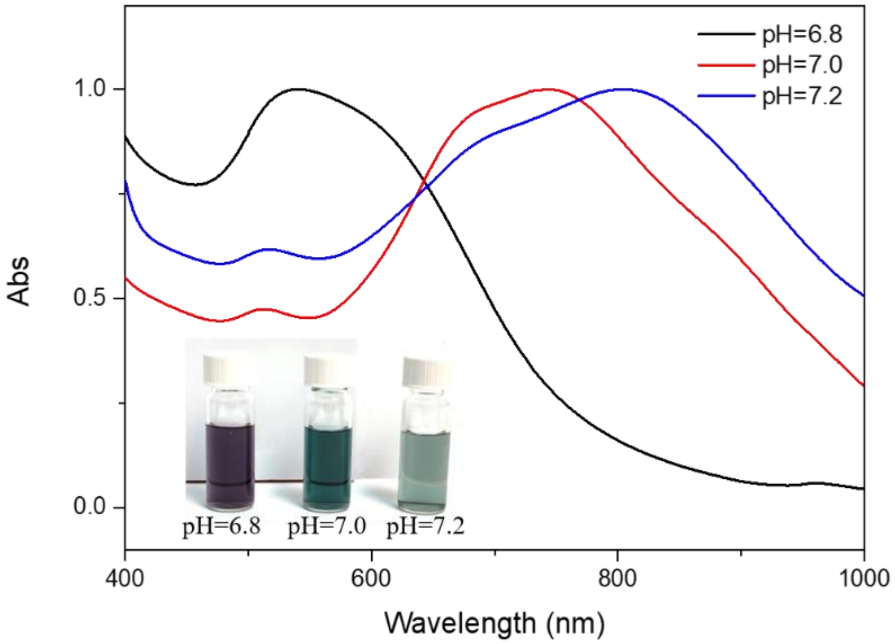

Figure S1. The absorption spectra of the three kinds of different HEPES buffer pH values.

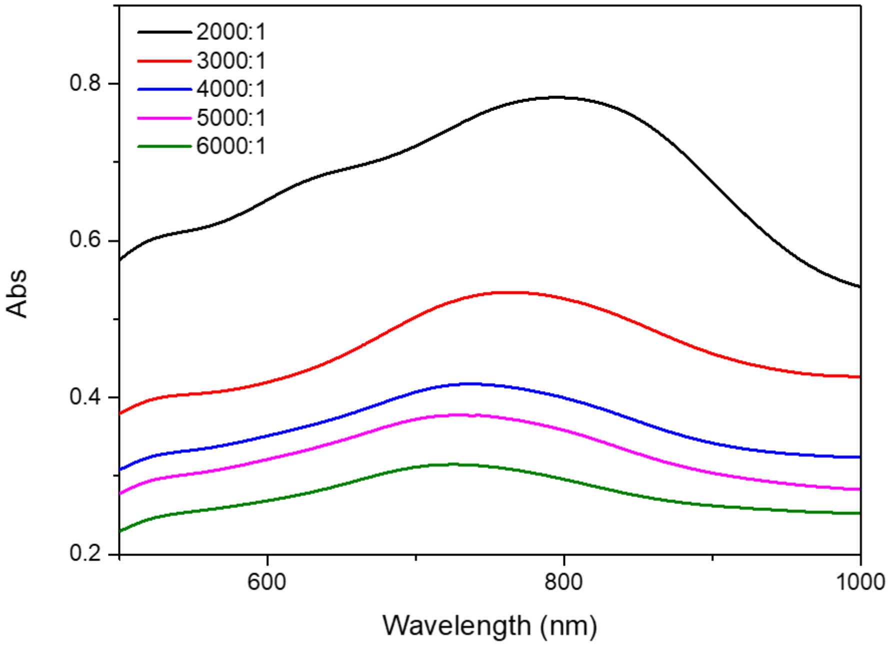

Figure S2. The absorption spectra of the different ratios of HEPES to $HAuCl_4$.

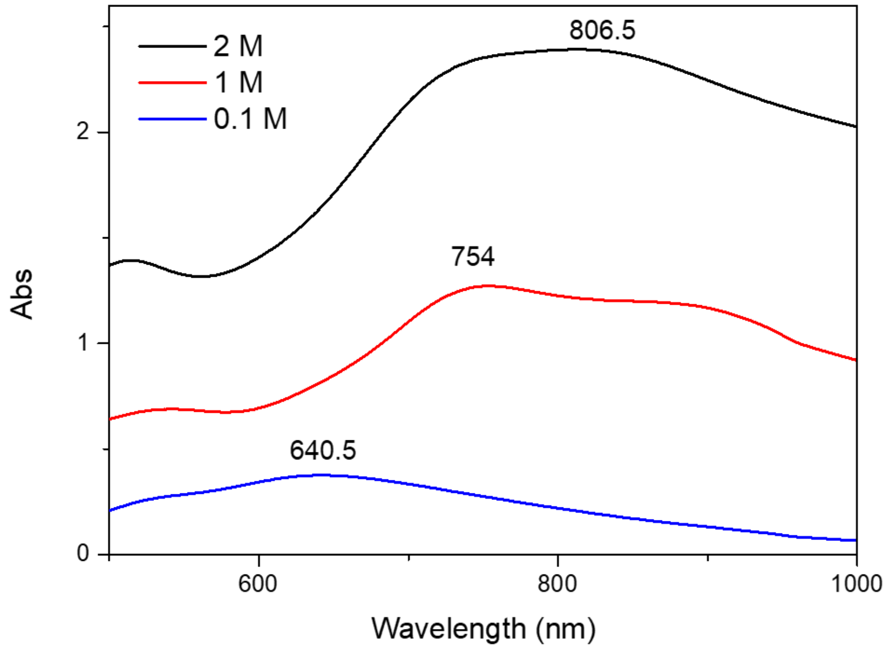

Figure S3. The absorption spectra of the different concentration of HEPES.

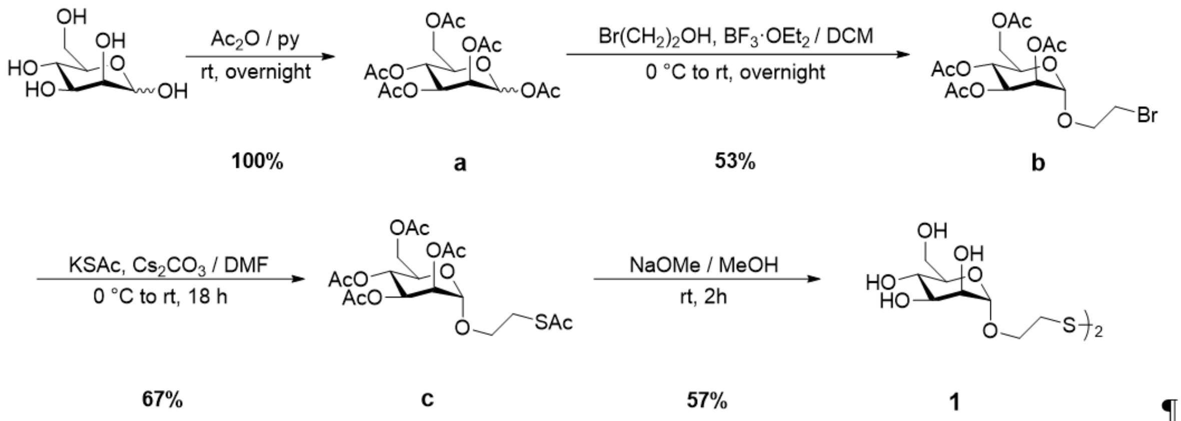

Scheme S1. The synthetic procedure of mannoside **1**.

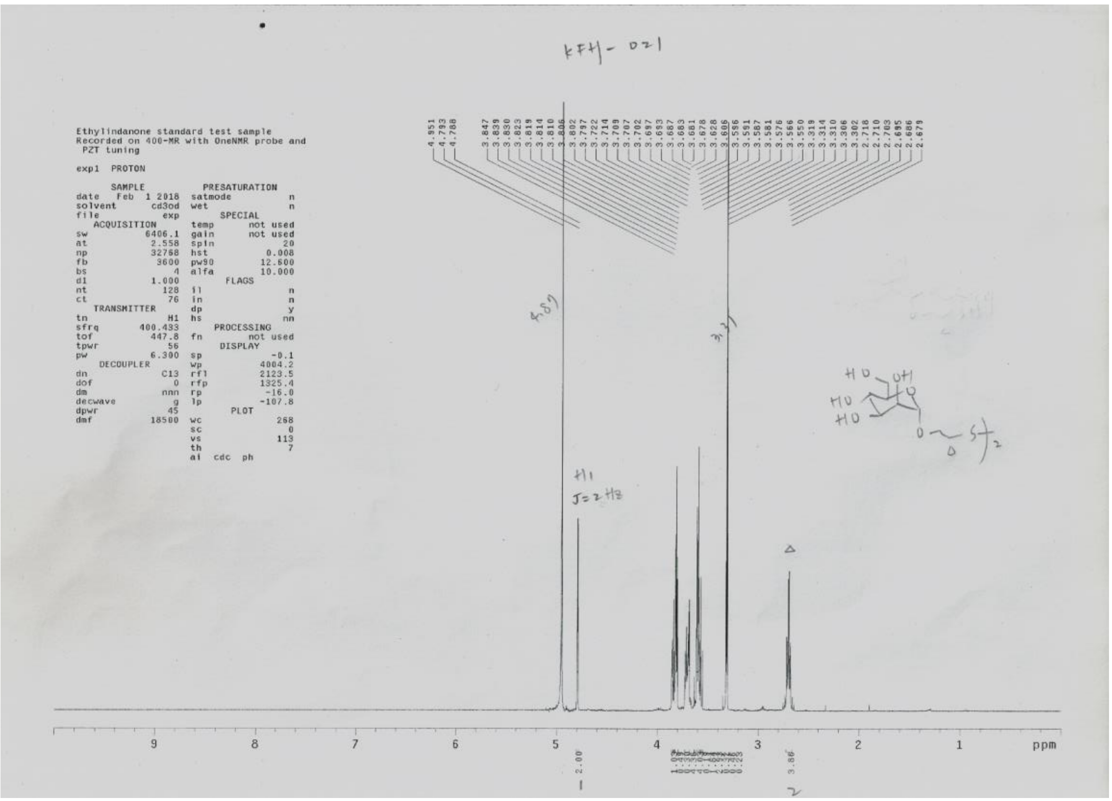

Figure S4. The NMR of thio-mannoside **1**.

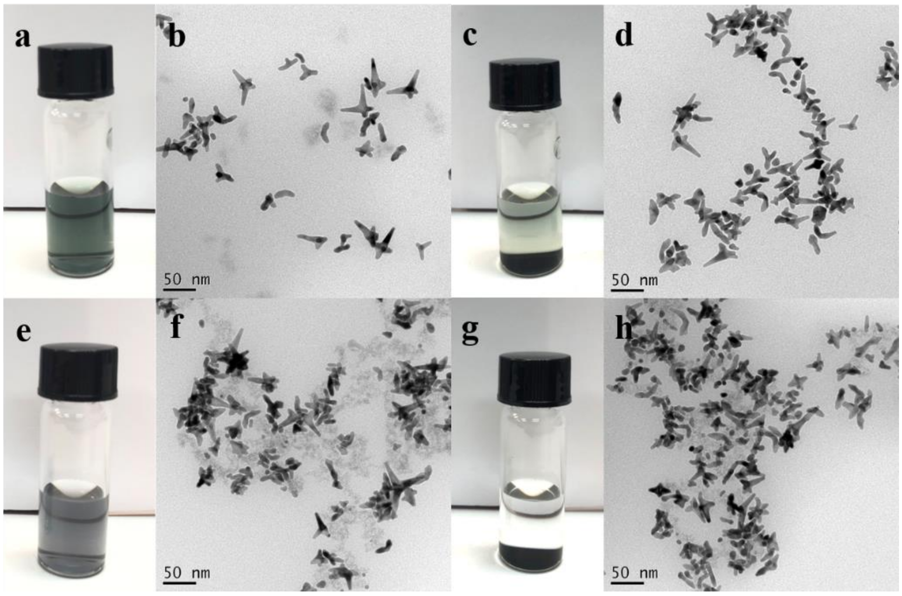

Figure S5. (a, b) Shows the fresh prepared solution of BAu and TEM images and (c, d) was 2 days after. The average size change from ~ 18.7 nm to ~ 17.5. (e, f) shows the the fresh prepared solution of Man@BAu and TEM images and (g, h) was 2 days after. The average size maintains at ~ 16 nm.

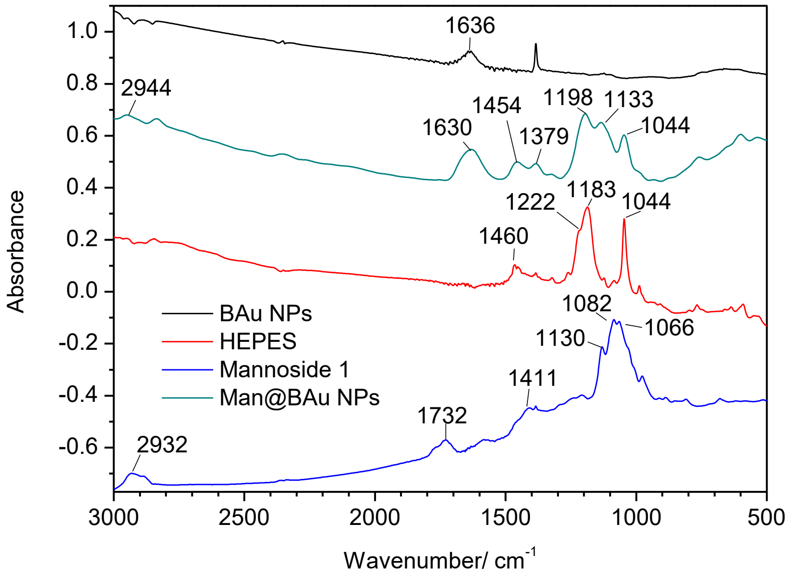

Figure S6. FTIR spectrum of (a) BAu and (b) Man@BAu NPs.

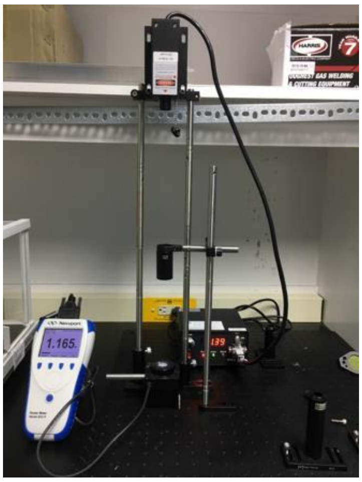

Figure S7. The 808 nm laser instrument.

To obtain the value of hS, a dimensionless driving force temperature, θ, was introduced using the maximum temperature of the system.

$$\theta = \frac{(T - T_{surr})}{(T_{max} - T_{surr})} \tag{S1}$$

The cooling time t and θ were obtained by the following eq 2.

$$t = -\tau_s ln(\theta) \tag{S2}$$

The time constant (τ_s) for heat transfer from the system could be determined by plotting linear time data from the cooling period against negative natural logarithm of driving force temperature (Figure S8b). Thus, τ_s is calculated to be 315.49 s.

$$hS = \frac{mC}{\tau_s} \tag{S3}$$

where m and C are the mass and heat capacity of water, respectively. The *hS* was determined to be 13.5 mW/ °C according to eq 3.

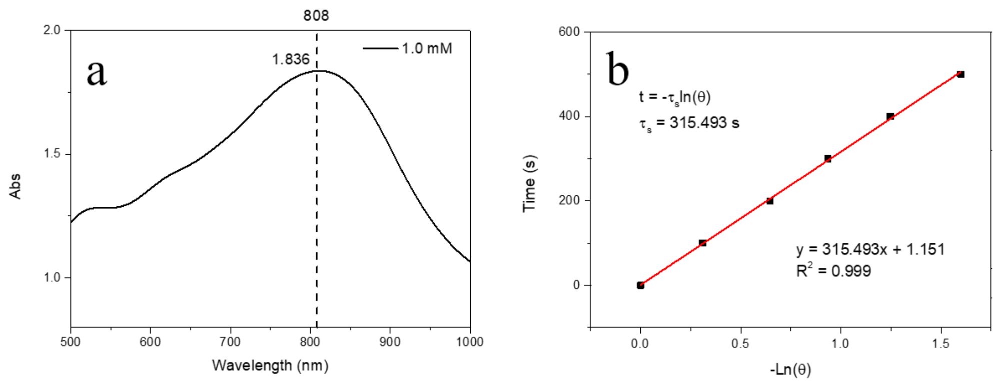

Figure S8. (a) UV absorption spectrum of Man@BAu at the concentration of 1.0 mM. The dash line is absorbance value at 808 nm. (b) A linear plot of time (after 600 s) versus negative natural logarithm of dimensionless driving force temperature obtained from the cooling stage of curve.

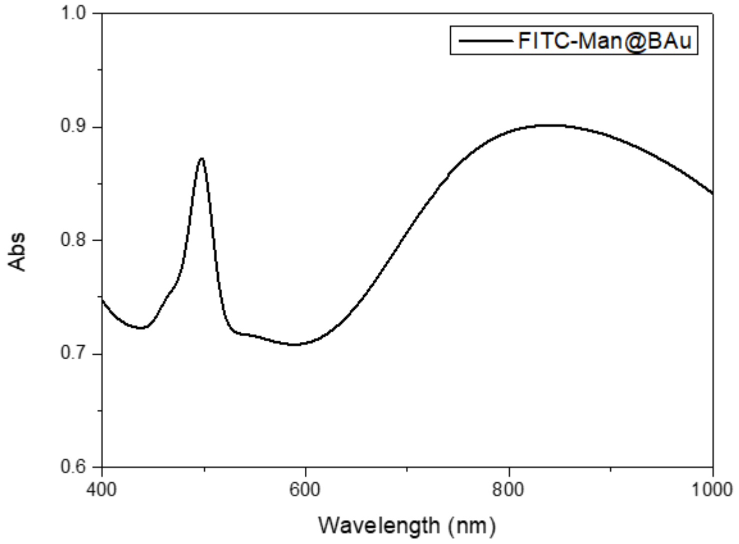

Figure S9. The UV spretum of FITC-Man@BAu NPs.
